# Combination of ultrasound and serological tests for detecting occult lateral lymph node metastases in medullary thyroid cancer

**DOI:** 10.1002/cam4.5856

**Published:** 2023-04-02

**Authors:** Jianchun Xiao, Jingyu Jiang, Weijie Chen, Tao Hong, Binglu Li, Xiaodong He, Wei Liu

**Affiliations:** ^1^ Department of General Surgery, Peking Union Medical College Hospital Chinese Academy of Medical Sciences, Peking Union Medical College Beijing China; ^2^ School of Medicine University of Tsinghua Beijing China; ^3^ Department of Surgery, Peking Union Medical College Hospital Chinese Academy of Medical Sciences Beijing China

**Keywords:** calcitonin, carcinoembryonic antigen, diagnostic study, lateral lymph node metastasis, medullary thyroid cancer, serum marker, ultrasound

## Abstract

**Purpose:**

To investigate the value of ultrasound and serum marker tests in detecting lateral lymph node metastasis in medullary thyroid cancer (MTC).

**Methods:**

Data of 105 patients diagnosed with MTC and admitted to the Department of General Surgery in Peking Union Medical College Hospital from June 2010 to August 2020 were collected and retrospectively analyzed.

**Results:**

Ultrasound examination alone had a sensitivity of 89.36% and a specificity of 70.69%. For surveillance of postoperative carcinoembryonic antigen and calcitonin, cut‐off values of 7.115 ng/mL and 13.185 pg/mL, respectively, were shown to discriminate the presence of cervical lymph node metastasis. Combining ultrasound and postoperative serum levels of both carcinoembryonic antigen and calcitonin as serial tests increased the specificity to 91.38% and 87.93%, with a sensitivity of 95.45%. Multivariate logistic analysis identified the following risk factors for lateral lymph node metastasis in MTC: suspicious lymph nodes detected by ultrasound and postoperative calcitonin above 13.185 pg/mL.

**Conclusion:**

The combination of ultrasound and serological tests achieved higher sensitivity and specificity to identify MTC cases with potential occult lateral cervical lymph node metastasis compared with single tests.

## Graphical abstract

The combination of ultrasound and serological tests achieved higher sensitivity and specificity to identify medullary thyroid cancer cases with potential occult lateral cervical lymph node metastasis compared with single tests. Surgeons should pay attention if a MTC patient has suspicious cervical sonographic results and CEA above 7.115 ng/ml and/or calcitonin above 13.185 pg/ml during early follow‐up, especially approximately 1 month after the surgery.

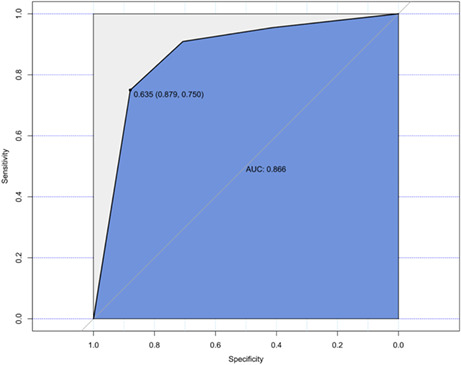

## 1 INTRODUCTION

Medullary thyroid cancer (MTC) is a rare malignancy, making up 1%–2% of the total thyroid malignancies in the United States, that originates from C‐cells in the thyroid.^1^ Because of the neuroendocrine feature of C‐cells, symptoms of MTC include palpitations, flushing, and diarrhea. If the tumor extends beyond the thyroid tissue and invades other cervical structures such as the laryngeal recurrent nerve and the airway, patients can experience hoarseness and dyspnea. MTC occurs sporadically, as sporadic MTC, or hereditarily, as a component of the type 2 multiple endocrine neoplasia syndrome (MEN2) or familial MTC. Sporadic MTC shows a high rate of cervical lymph node metastasis, with metastasis rates of 14% and 11% for central and lateral compartments in patients with T1 stage, respectively; in patients with T4 stage, these rates are 86% and 93%.^2^ Apart from surgical treatment for MTC, numerous efforts have also been devoted to identifying novel targets for treating MTC. For example, inhibiting the aurora kinases supresses the in vitro growth of MTC derived cell lines^3^, ^4^ and there have been several clinical trials exploring the effectiveness of aurora kinase inhibitors in treating various tumors.^5^, ^6^ Moreover, blocking aurora kinase was shown to be useful for suppressing both papillary^7^ and anaplastic thyroid cancer^8^, ^9^ in vitro, indicating the potential usefulness of these agents in treating thyroid malignancies.

Calcitonin and carcinoembryonic antigen (CEA) are the two most common serum markers for MTC. Total thyroid excision with surgical removal of cervical lymph node compartment is the standard treatment for both sporadic and hereditary MTC,^1^, ^10^ according to the ultrasound results and serum calcitonin level preoperatively. While the lateral lymph node metastasis (LLNM) rate is high in MTC cases, whether prophylactic lateral lymph node dissection is beneficial for MTC patients is unclear. Furthermore, while nodal metastasis is a risk factor for poor prognosis in MTC, there are currently no effective methods for preoperative assessment of LLNM.^11^, ^12^ One study showed that the preoperative calcitonin level is associated with the extent of tumor and the number of lateral lymph node metastases in MTC (*n* = 170); thresholds of 20.1, 200.1, and 500.1 pg/mL were identified for ipsilateral LLNM, contralateral LLNM, and distant metastasis, respectively,^13^ while 20 and 200 pg/mL were recommended by other doctors for ipsilateral and contralateral prophylactic lymph node dissection, respectively.^1^, ^14^ However, the role of preoperative calcitonin in predicting LLNM remains unclear. In another retrospective study,^15^ 16%, 50%, and 71% patients with LLNM had preoperative calcitonin thresholds below 500, 501–1000, or above 1000 pg/mL, respectively; among patients without distal metastases or disease progression, 19% of N0 cases had calcitonin above 500 pmol/L and 17% of N1b cases had calcitonin ≤500 pmol/L. Moreover, multivariate analyses identified tumor extension beyond the thyroid (*p* = 0.007) and failure to reach biochemical cure (*p* = 0.028), but not basal calcitonin levels, as risk factors for LLNM. High levels of preoperative CEA were also indicative of number of lymph nodes with metastases (*r* = 0.47), while basal calcitonin showed a better correlation (*r* = 0.59).^14^ The study by Fan et al. suggests that lateral neck dissection should be considered for patients with thyroid capsular invasion or high preoperative CEA, especially when CEA reaches 30 ng/mL, which indicates a high possibility of central nodal metastases.^16^ A retrospective study of 233 patients identified that tumor size over 4 cm, extension beyond the thyroid and distant metastasis independently contribute to MTC‐related death,^17^ and extrathyroid extension correlated with distal metastasis.^17^ Preoperative calcitonin above 65 pg/mL, tumor larger than 1.5 cm and three sonographic features of the tumor (irregular shape, speculated margin, and subcapsular location) were reported as predictors for LLNM in another study,^18^ which suggests that prophylactic lateral neck dissection is not necessary for patients with fewer than two predictors. The study by Wu et al.^19^ suggests prophylactic lateral lymph node removal for patients with positive central lymph nodes and/or lateral lymph nodes by sonography.

Few studies have reported the effectiveness of ultrasound and serological tests for CEA and calcitonin, either as single or combined tests, in the evaluation of LLNM for MTC. Therefore, in this study, we compared the effectiveness of ultrasound and serological tests by retrospectively reviewing the medical history of 105 MTC patients treated at our center. We further investigated risk factors for LLNM in MTC patients that may distinguish patients who may need a lateral lymph node dissection.

## 2 METHODS

### 2.1 Ethics

Prior to the surgical procedures, written informed consent was provided by all patients or legal guardians for potential academic usage of their medical records. This study was approved by the ethical committee of Peking Union Medical College Hospital (PUMCH).

### 2.2 Patients

We retrieved the medical records of patients who were diagnosed with MTC and admitted to the Department of General Surgery in PUMCH from June 2010 to August 2020. The included patients met the following criteria: (i) postoperative confirmation of MTC diagnosis by pathology, using hematoxylin–eosin staining, with immunohistochemical validation when necessary, (ii) patients who underwent primary surgery or radical repeat‐surgery at our hospital with complete color Doppler ultrasound records at PUMCH, and (iii) no previous diagnosis of malignancies other than MTC. The exclusion criteria were as follows: (i) patients with a primary neoplasm occurring outside the thyroid, (ii) patients who were transferred to our department without detailed medical records, and (iii) patients who did not have postoperative calcitonin results or ultrasonic examinations at our hospital.

A total of 105 patients were included in this retrospective study. At the time of surgery, 47 patients had lateral cervical lymph node metastases as determined by intraoperative frozen sections or postoperative pathology (the LLNM+ group) and 58 patients were pathologically negative for lateral lymph node metastases (the LLNM− group). All LLNM− patients underwent cervical ultrasonic examinations at 3 months and 6 months postoperatively to exclude potential nodal metastases. The follow‐up time was calculated as the duration between the time of surgery at our department and the last MTC‐specific follow‐up. The median follow‐up time was 1005 days. We used the serological tests conducted at the first postoperative MTC‐specific follow‐up at the outpatient department, with a median time of 35 days after the surgery.

### 2.3 Sonography and laboratory tests

**FIGURE 1:**
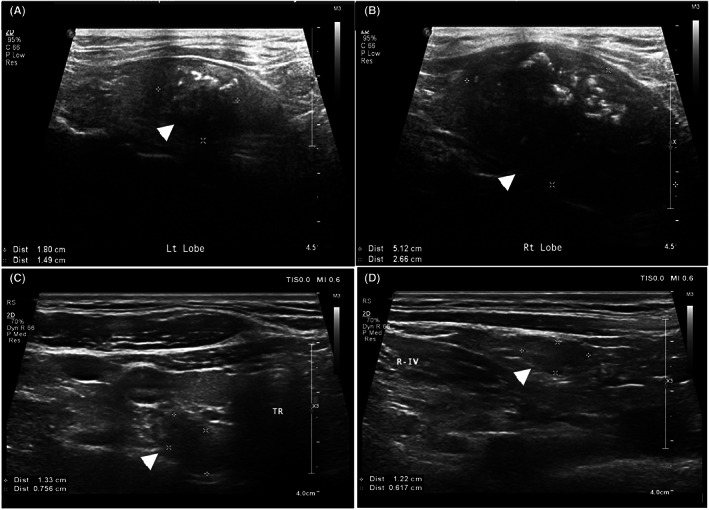
Characteristic ultrasonic images of MTC tumors and lymph nodes. (A) Longitudinal section of a hypoechogenic tumor located in the left lobe, with irregular contour, ill‐defined margin and internal calcifications (arrowhead). (B) Another hypoechogenic lesion with similar features identified in the right lobe of the same patient (arrowhead). (C) Suspicious lymph node (arrowhead) detected in the right central compartment near the trachea (TR), with unclear boundary between the nodal cortex and medullary. (D) Enlarged lymph node with internal calcification detected in region IV of the right neck (R‐IV), as indicated by the arrowhead.

Ultrasound examination was conducted by sonographers at PUMCH. The ultrasound features of suspicious cervical lymph nodes and thyroid nodules, that is, echo density, longitudinal and transverse diameters, calcification, internal cysts, boundary, and color‐flow doppler imaging (CDFI) for blood flow detection, were taken using Phillips IU22 (Philips Healthcare, Eindhoven, Netherlands) and GELogiq9 (GE Healthcare) scanners. As the determination of irregular shape and loss of the lymph node hilar structure or cortical‐medullary border depend on the subjective evaluation from the sonographer, they were not included in the analysis. The normal value of serological calcitonin at our hospital is below 10 pg/mL (detectable range: 1.5–1535 pg/mL). For CEA, the normal value is below 5 ng/mL. The characteristic features of the tumors and suspicious lymph nodes are illustrated in Figure 1.

### 2.4 Statistical analysis

The distribution of data was examined by the Shapiro–Wilk test. Normally distributed data were compared using Student's *t‐*test and are presented as mean ± standard deviation. For variables with skewness, the mean, median, and 25th to 75th percentiles were examined by the Mann–Whitney *U*‐test. For qualitative comparison, Fisher's exact test or the chi‐square test were used when appropriate. Variables with a *p*‐value less than 0.1 from univariate tests were subjected to logistic multivariate regression analysis. Receiver operating characteristic (ROC) curves were generated to determine the best cut‐off value of serum marker levels by calculating the Youden index. To evaluate the prediction effectiveness of the logistic multivariate model, the area under the curve (AUC) of the ROC curve was calculated. The Statistical Package for Social Sciences software (version 25.0, IBM Corp.) was used for data analyses, and the R package pROC was hired to generate the plots.^20^, ^21^ A *p*‐value less than 0.05 was considered statistically significant.

## 3 RESULTS

### 3.1 Clinical features

This study included 105 patients who were diagnosed with MTC and admitted to the Department of General Surgery in PUMCH from June 2010 to August 2020. Among the total 105 patients, 47 were LLNM positive (the LLNM+ group) and 58 patients did not have LLNM (the LLNM− group), as validated by intraoperative frozen sections and/or postoperative pathology.

There were no differences between groups regarding the distribution of age, sex, MEN mutation frequency, comorbidities, and thyroid nodule multifocality (Table 1). The LLNM+ group had significantly more cases with multiple suspicious lymph nodes (*p* = 0.004).

**TABLE 1:** General characteristics of both groups.

Variable	LLNM+ group (*n* = 47)	LLNM− group (*n* = 58)	Value	*p*‐value
Age(years)	48.426 ± 11.286	47.397 ± 12.259	*t* = 0.443	0.695
Gender				
Male	23	19	χ^2^ = 2.2	0.138
Female	24	39		
MEN 2a /2b	2	5	χ^2^ = 0.25	0.617
Comorbidities				
HTN	8	12	χ^2^ = 0.05	0.823
DM	4	8	χ^2^ = 0.29	0.590
Multiple LN enlargement	20	9	χ^2^ = 8.19	0.004
Multifocal thyroid nodules	17	33	χ^2^ = 3.68	0.055

Abbreviations: DM, Diabetes mellitus; HTN, hypertention; LLNM, lateral lymph node metastases; LN, lymph node; MEN, multiple endocrine neoplasia.

Among the 47 LLNM+ patients, 42 patients had enlarged or swelling cervical lymph nodes as shown by ultrasound examinations preoperatively; 30 patients had suspicious lateral compartment on ultrasound and 5 LLNM+ patients had normal preoperative ultrasonic examination regarding both central and lateral lymph nodes and were diagnosed by intraoperative assessment. In comparison, only 17 patients from the LLNM− group had suspicious lymphadenopathy detected by ultrasound preoperatively. Among these 17 patients, 2 had lateral lymph node enlargement on ultrasonography.

In the overall patient group, 97 patients had their primary surgery at our hospital, and the other 8 patients underwent re‐surgery for LLNM or dissection of the residual lobe with lateral lymph nodes. Among the 58 patients in the LLNM− group, 20 patients with suspicious ultrasonic lymph node enlargement, abnormal intraoperative frozen sections, or intraoperative clinical assessment received total thyroidectomy with central and lateral lymph node dissection. The other 38 LLNM− patients received radical surgery for thyroid cancer with removal of the central lymph node compartment. Only several patients complained of symptoms, such as neck discomfort (*n* = 7), and palpitation (*n* = 1).

### 3.2 Characterization of the sonographic features of both groups

Information of 59 and 26 suspicious lymph nodes was retrieved from the ultrasound examination reports for the LLNM+ group and LLNM− group, respectively. The frequencies of the following features were compared between groups: hypoecho or extreme hypoecho, unclear boundary, blood flow around, or within the lymph node detected by CDFI, macrocalcification and cysts within the lymph node, all of which are considered to be of value for indicating lymph node metastases. As shown in Table 2, the LLNM+ group had more lymph nodes with an unclear boundary (*p* = 0.021) and positive CDFI (*p* = 0.009) than the LLNM− group.

**TABLE 2:** Comparison of the ultrasonic features of the lymph nodes detected by ultrasound in both groups.

Ultrasound features	LLNM+ group (*n* = 59)	LLNM− group (*n* = 26)	χ2	*p*‐value
Echo				
Hypoecho/extreme hypoecho	48	26	0.11	0.740
Isoecho/hyperecho	1	0		
Boundary				
Clear	21	17	5.33	0.021
Unclear	38	9		
Blood flow				
CDFI (−)	15	15	6.88	0.009
CDFI (+)	44	11		
Calcification				
Calcification (−)	45	22	0.34	0.560
Calcification (+)	14	4		
Internal cyst				
Cyst (−)	49	25	0.11	0.740
Cyst(+)	0	1		

Abbreviation: CDFI, color‐flow doppler imaging.

Metastatic lymph nodes usually have rounded shape, as do malignant thyroid nodules. Therefore, we compared the sonographic diameters of both lymph nodes and nodules between the two groups. As shown in Table 3, in the LLNM+ group, the L/T ratio (defined as lymph node longitudinal diameter/ transverse diameter) was significantly lower than that of the LLNM− group (*p* = 0.001). The LLNM+ patients had a significantly larger lymph node transverse diameter (*p* = 0.005), tumor longitudinal diameter (*p* = 0.008) and tumor transverse diameter (*p* = 0.03) than the LLNM− patients. The cut‐off values for the lymph node L/T ratio, L diameter, and T diameter were 2.07 (sensitivity: 88.9%, specificity: 67.7%), 0.45 cm (sensitivity: 88.6%, specificity: 74.1%), and 0.25 cm (sensitivity: 88.6%, specificity: 75.9%), respectively.

**TABLE 3:** Comparison of the sonographic diameters between both groups.

	LLNM+ group (mean, median, 25%–75%)	LLNM− group (mean, median, 25%–75%)	*Z*‐value	*p*‐value
LN L diameter (cm)	1.43, 1.30, 0.90–1.80	1.52, 1.40, 1.00–2.13	0.73	0.465
LN T diameter (cm)	0.77, 0.70, 0.50–0.900	0.60, 0.45, 0.38–0.63	−2.812	0.005
LN L/T ratio	2.04, 1.80, 1.44–2.25	3.09, 2.68, 1.84–4.21	3.297	0.001
Tumor L diameter (cm)	2.07, 1.50, 1.10–2.60	1.52, 1.10, 0.70–2.00	−2.636	0.008
Tumor T diameter (cm)	1.53, 1.10, 0.80–1.90	1.14, 0.90, 0.60–1.50	−2.168	0.030
Tumor L/T ratio	1.39, 1.27, 1.17–1.59	1.36, 1.25, 1.06–1.50	−1.123	0.261

Abbreviations: L diameter, longitudinal diameter; L/T ratio, lymph node longitudinal diameter/transverse diameter; T diameter, transverse diameter.

### 3.3 Laboratory test features of both groups

In the LLNM+ group, 26 patients had preoperative results for serum calcitonin and 27 had results for serum CEA, and 17 and 25 had these data in the LLNM− group, respectively. All patients had postoperative calcitonin values. Postoperative CEA results were available for 45 and 51 patients from the LLNM+ and LLNM− groups, respectively. Both postoperative calcitonin (*p* = 0.001) and CEA (*p* = 0.002) levels were significantly higher in the LLNM+ group, while there was no statistical difference in preoperative calcitonin (*p* = 0.237) or CEA (*p* = 0.097) between the two groups (Table 4). We calculated the AUC of the ROC curves and identified cut‐off values of 7.115 ng/mL and 13.185 pg/mL for postoperative CEA and calcitonin, respectively, to discriminate between the two groups. The cut‐off values for preoperative CEA and calcitonin are 80.75 ng/mL and 904.515 pg/mL, respectively.

**TABLE 4:** Comparison of the serum CT and CEA levels between both groups.

	LLNM+ group (mean, median, 25%–75%)	LLNM− group (mean, median, 25%–75%)	*Z*‐value	*p*‐value
Preoperative calcitonin (pg/mL)	842.46, 476.61, 247.159–1490.92	536.84, 261.68, 121.20–792.49	−1.184	0.237
Postoperative calcitonin (pg/mL)	222.37, 33.82, 17.77–150.39	84.61, 7.31, 1.50–45.42	−3.464	0.001
Preoperative CEA (ng/mL)	107.34, 49.94, 12.60–139.80	64.09, 22.30, 6.96–57.25	−1.658	0.097
Postoperative CEA (ng/mL)	29.22, 9.05, 4.17–28.31	21.05, 4.28, 1.79–8.63	−3.142	0.002

### 3.4 Multivariate Logistic Regression

**FIGURE 2:**
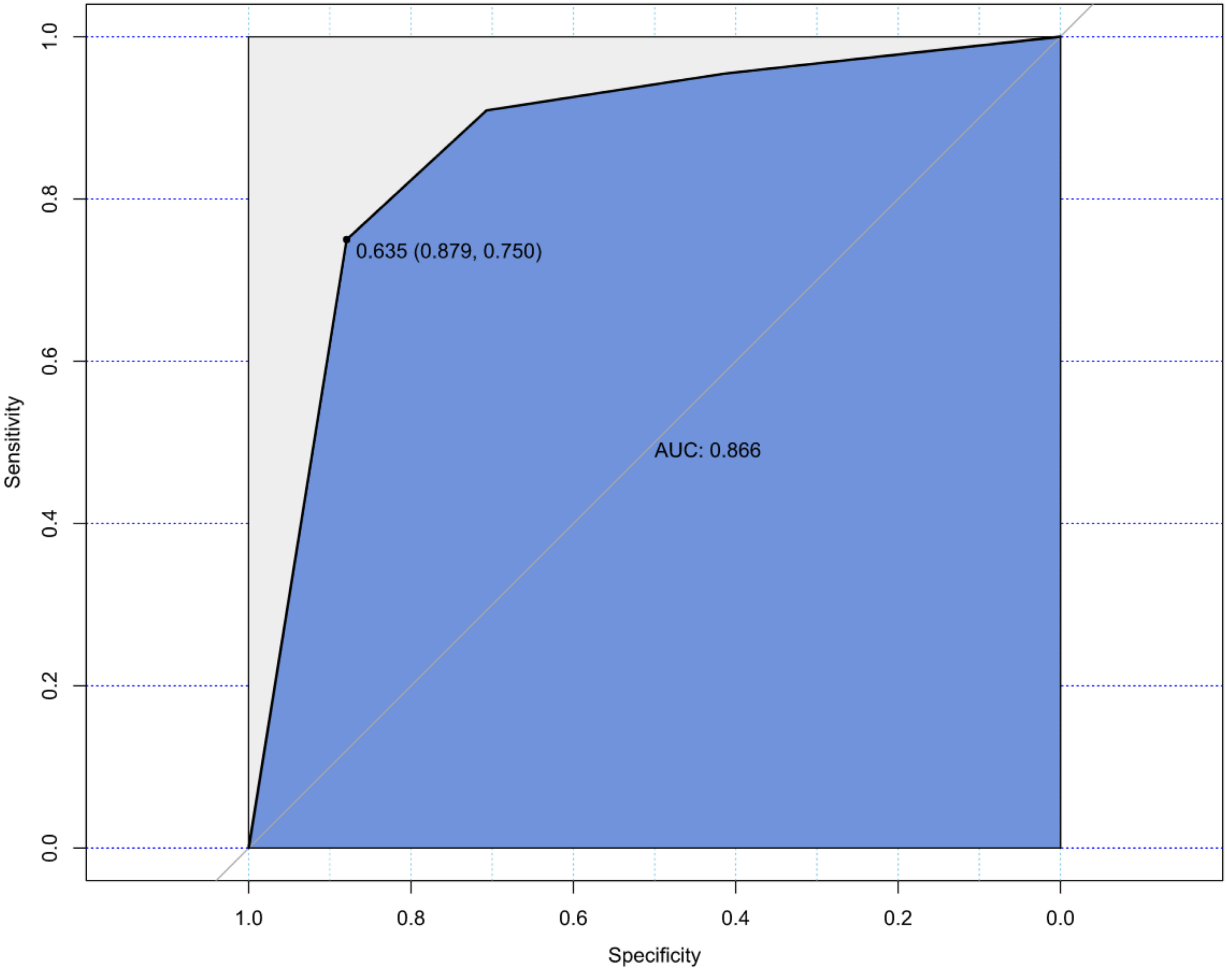
ROC curve for predicting cervical LN metastasis according to the logistic model, with an AUC of 0.886 (95% CI: 0.791–0.904).

Variables with a *p*‐value less than 0.1 from the preceding analyses (multiple suspicious lymph nodes, multifocal thyroid nodules, unclear LN boundary, LN blood flow, LN L/T ratio (lymph node longitudinal diameter/ transverse diameter), tumor L diameter, tumor T diameter, raised postoperative CEA and calcitonin), and suspicious sonograph result for lateral lymph node enlargement were subjected to binary logistic analysis. Two risk factors were identified: suspicious sonographic results of cervical lymph nodes and postoperative calcitonin above 13.185 pg/mL (Table 5). The AUC of the ROC curve for this model was 0.866 (Figure 2).

**TABLE 5:** Logistic analysis results for LN metastasis in MTC patients.

	OR, 95% CI	*p*‐value
Ultrasound reporting suspicious LN	21.600, 6.405–72.843	<0.001
Postoperative calcitonin > 13.185 pg/mL	4.540, 1.531–13.460	0.006

### 3.5 Combination of Sonographic and Serum Tests for Predicting LLNM

We calculated the specificity, sensitivity, positive predictive value (PPV), negative predictive value (NPV), positive likelihood ratio (LR+), and negative likelihood ratio (LR−) for sonographic and serum tests and their combinations. Table 6 and Table 7 list the values for the sonographic and preoperative or postoperative serum tests. Preoperative calcitonin value above 904.515 pg/mL shows a low sensitivity (41.67%) but high specificity (82.35%), while postoperative calcitonin higher than 13.185 pg/mL has a higher sensitivity (75.90%) but lower specificity (58.62%). Ultrasound alone has a sensitivity of 89.36% and a specificity of 70.69%. When parallelly combined with preoperative or postoperative calcitonin, similar values of sensitivity were reached (95.83% and 95.45%, respectively). Together, these results indicate that the combination of ultrasound with serological tests shows a specificity above 85% both preoperatively and postoperatively, while parallel combinations all have sensitivity above 95%.

**TABLE 6:** Single and combined diagnostic parameters of ultrasound, preoperative serum CEA and calcitonin.

	Sensitivity%	Specificity%	PPV%	NPV%	LR+	LR−
Ultrasound	89.36,76.11–96.02	70.69, 57.09–81.54	71.19, 57.73–81.87	89.13, 75.64–95.93	3.05, 2.02–4.60	0.15, 0.06–0.35
CEA^a^	44.00, 25.02–64.73	88.00, 67.66–96.85	78.5, 48.82–94.30	61.11, 43.53–76.38	3.67, 1.16–11.58	0.63, 0.44–0.91
calcitonin ^b^	41.67, 22.80–63.06	82.35, 55.80–95.33	76.92, 45.98–93.84	50.00, 31.07–68.93	2.36, 0.76–7.31	0.71, 0.49–1.02
Ultrasound & CEA^c^	36.00, 18.71–57.38	92.00, 72.50–98.60	81.82, 47.76–96.79	58.97, 42.19–74.02	4.5, 1.08–18.77	0.70, 0.51–0.94
Ultrasound|CEA^d^	96.00, 77.68–99.79	72.00, 50.40–87.13	77.42, 58.46–89.72	94.74, 71.89–99.72	3.43, 1.82–6.46	0.06, 0.01–0.39
Ultrasound & calcitonin^e^	33.33, 16.43–55.31	94.12, 69.24–99.69	88.89, 50.67–99.42	50.00, 32.24–67.76	5.67, 0.78–41.20	0.71, 0.53–0.95
Ultrasound|calcitonin^f^	95.83, 76.88–99.78	58.82, 33.45–80.57	76.67, 57.30–89.37	89.36, 57.12–99.52	2.33, 1.31–4.13	0.07, 0.01–0.52

*Note*: Values are presented as estimated value, 95% CI.

^a^ Preoperative CEA > 80.75 ng/mL.

^b^ Preoperative calcitonin > 904.515 pg/mL.

^c^ Ultrasound and preoperative serum CEA test in serial.

^d^ Ultrasound and preoperative serum CEA test in parallel.

^e^ Ultrasound and preoperative serum calcitonin test in serial.

^f^ Ultrasound and preoperative serum calcitonin test in parallel.

**TABLE 7:** Single and combined diagnostic parameters of ultrasound, postoperative serum CEA and calcitonin.

	Sensitivity%	Specificity%	PPV%	NPV%	LR+	LR−
Ultrasound	89.36,76.11–96.02	70.69, 57.09–81.54	71.19, 57.73–81.87	89.13, 75.64–95.93	3.05, 2.02–4.60	0.15, 0.06–0.35
CEA^a^	63.64, 47.74–77.17	72.41, 58.89–82.95	63.64, 47.74–77.17	72.41, 58.89–82.95	2.31, 1.44–3.70	0.50, 0.34–0.75
calcitonin ^b^	79.55, 64.25–89.67	58.62, 44.96–71.14	59.32, 45.76–71.67	79.07, 63.52–89.42	1.92, 1.37–2.70	0.35, 0.19–0.54
Ultrasound and CEA^c^	59.09, 43.31–73.30	91.38, 80.28–96.78	83.87, 65.53–93.91	74.65, 62.69–83.90	6.85, 2.86–16.41	0.45, 0.31–0.64
Ultrasound|CEA^d^	95.45, 83.30–99.21	51.72, 38.35–64.87	60.00, 47.59–71.31	93.75, 77.78–98.91	1.98, 1.50–2.60	0.09, 0.02–0.35
Ultrasound and calcitonin^e^	75.00, 59.35–86.30	87.93, 76.09–94.61	82.50, 66.64–92.11	82.26, 70.05–90.40	6.21, 3.04–12.71	0.28, 0.17–0.48
Ultrasound|calcitonin^f^	95.45, 83.30–99.21	41.38, 28.86–55.04	55.26, 43.46–66.53	92.31, 73.40–98.66	1.63, 1.30–2.04	0.11, 0.03–0.45
Ultrasound and calcitonin and CEA^g^	52.27, 36.88–67.27	93.10, 82.45–97.77	85.19, 65.39–95.14	72.00, 60.26–81.46	7.58, 2.83–20.33	0.51, 0.38–0.70
ultrasound|calcitonin|CEA^h^	95.45, 83.30–99.21	36.21, 24.31–49.94	53.16, 41.66–64.36	91.30, 70.49–98.48	1.50, 1.22–1.84	0.13, 0.03–0.52

*Note*: Values are presented as estimated value, 95% CI.

^a^ Postoperative CEA > 7.115 ng/mL.

^b^ Postoperative calcitonin > 13.185 pg/mL.

^c^ Ultrasound and postoperative serum CEA test in serial.

^d^ Ultrasound and postoperative serum CEA test in parallel.

^e^ Ultrasound and postoperative serum calcitonin test in serial.

^f^ Ultrasound and postoperative serum calcitonin test in parallel.

^g^ Ultrasound, post operative calcitonin, and postoperative serum CEA test in serial.

^h^ Ultrasound, post operative calcitonin, and postoperative serum CEA test in parallel.

## 4 DISCUSSION

In this study, we investigated the features of sonographic and laboratory tests for 47 MTC cases with LLNM and found that there is a higher possibility for hypoecho or extreme hypoecho thyroid nodules with larger longitudinal diameters in such cases. Moreover, univariate analysis revealed that these patients tend to have more lymph nodes with an enlarged size, rounded shape, unclear boundary and abnormal blood flow detected by CDFI.

We used the ultrasonic finding of central and/or lateral lymph node enlargement for the calculation of sensitivity and specificity, as both central lymph node metastasis and positive lateral lymph node on ultrasound are independent risk factors for LLNM in MTC.^19^ The sensitivity and specificity for a single ultrasound test are 89.36% and 70.69%, respectively. Previous work reported a high specificity (central: 95%, lateral 88%) but low sensitivity (central: 28.4%, lateral 75.8%) when using ultrasound to detect nodal metastases in papillary thyroid cancer.^22^ A possible explanation is that central nodal metastases is the most common form in papillary thyroid cancer; its detection requires skilled sonographers because the existence of anatomic structures like the airway can hinder the detection of lymph nodes by ultrasound, leading to a much lower sensitivity in the central compartment than the lateral compartment. Another study reported a lower sensitivity (central: 6%, lateral 56%, overall 43%) but high specificity (central: 100%, lateral 97%, overall 97%) when using ultrasound to detect nodal metastases.^23^ One possible reason for this difference is that ultrasound examination relies on the sonographers' experience and subjective judgment, and thus false positive or false negative results are not uncommon. In accordance with the previous study, only two patients from the LLNM− group in our study had false positive lateral lymph nodes on ultrasound, suggesting that ultrasound has high specificity in distinguishing LLNM. To improve the detection rate of occult nodal metastases, both the central and lateral compartments should be taken into consideration.

Regarding the normalization of postoperative serum CEA and calcitonin, there is no consensus as to how long it takes for the markers to reach a nadir. Some studies suggest that 3 months may be a reasonable duration for calcitonin,^24^, ^25^ while other studies suggested that calcitonin should reach an undetectable nadir within 1 month postoperatively if the surgical procedure is curative.^26^ Postoperative CEA reached the nadir in approximately 63% of cured patients in 1 month and 98% patients by 6 months postoperatively.^26^ The American Thyroid Association (ATA) guideline currently recommends a serum test for calcitonin and CEA 3 months postoperatively (grade C); if results are in the normal range, follow‐ups can be carried out every 6 months for 1 year and then annually. If abnormal values are detected, especially a postoperative calcitonin above 150 pg/mL, imaging and physical examination should be additionally carried out to examine potential metastases. We used the serological results from the first postoperative follow‐up in the calculation, with a median time of 35 days after the surgery, and found that a cut‐off value of 7.115 ng/mL for postoperative CEA and 13.185 pg/mL for postoperative calcitonin are useful to distinguish the presence of LLNM in MTC patients. A previous study reported recurrence in cases with a normal postoperative calcitonin result and a CEA level as low as 9.96 ng/mL,^27^ indicating that patients with only elevated postoperative CEA should be suspected for MTC recurrence. Our data suggest that surgeons should be vigilant if the postoperative calcitonin or CEA reaches the indicated cut‐off values because there may be an underlying local recurrence or nodal metastasis, as most patients should reach a serological nadir at the time of follow‐up.

Determining the extent of neck dissection is crucial in the treatment of MTC. Our results are consistent with previous literature regarding the ultrasonic features of the tumor diameters, postoperative serological markers and the usefulness of ultrasound in detecting LLNM. Other prognostic factors were suggested by some studies, including male sex,^17^, ^28^ distal metastasis,^17^, ^28^ multifocality,^28^ and ratio of postoperative calcitonin to preoperative calcitonin,^29^ as a ratio above 0.15 indicates poorer prognosis. As reviewed by Ahn et al.^30^ there is a negative association between the frequency of ipsilateral lateral neck dissections and the rate of death caused by MTC (*p* = 0.0017), so careful consideration regarding the extent of surgery should be taken for the benefit of patients.

This study has several limitations. First, we have limited available data regarding the preoperative serological markers. Therefore, more data should be collected to calculate the rational cut‐off values for calcitonin and CEA preoperatively. Currently, the combination of postoperative serological marker levels and ultrasound examination shows a sensitivity of 95.45% by parallel tests and a highest specificity of 93.10% using postoperative calcitonin and CEA and ultrasound as a serial test. However, more research is needed for the preoperative prediction of LLNM. Second, the precise calculation of tumor size and detection of abnormal lymph nodes by ultrasound preoperatively remains a burden for sonographers, leading to great variance of the predictive value of ultrasonography for LLNM in MTC. Finally, this was a retrospective study at a single center, thus leading to potential selection bias. Multicenter randomized controlled studies are therefore required.

## 5 CONCLUSION

Our study revealed that the combination of ultrasound and postoperative surveillance of serological markers helps in identifying MTC patients with potential occult nodal metastases. Surgeons should pay attention if a MTC patient has suspicious cervical sonographic results and CEA above 7.115 ng/mL and/or calcitonin above 13.185 pg/mL during early follow‐up, especially approximately 1 month after the surgery, which suggests potential occult metastases to the lateral compartment lymph nodes and a requirement for surgical removal, if required. Randomized controlled studies are needed to identify rational parameters for predicting lateral nodal metastases preoperatively.

## AUTHOR CONTRIBUTIONS

JJ and JX designed the study, collected the data, and wrote the manuscript. JX revised the manuscript. All authors contributed to the editing process and approved the final version.

## FUNDING INFORMATION

This study was supported by Clinical Research Project of Peking Union Medical College Hospital (2022‐PUMCH‐A056), Youth Research Fund of Peking Union Medical College Hospital (pumch201910819) and the National Key Research and Development Program (Grant no. 2020YFF0305104).

## CONFLICT OF INTEREST STATEMENT

The authors declare no conflicts of interest.

## Data Availability

Data supporting this study will be available from the corresponding author upon reasonable request.
